# Concomitant *KRAS* mutations attenuate sensitivity of non-small cell lung cancer cells to KRAS G12C inhibition

**DOI:** 10.1038/s41598-022-06369-3

**Published:** 2022-02-17

**Authors:** Tereza Vaclova, Atanu Chakraborty, James Sherwood, Sarah Ross, Danielle Carroll, J. Carl Barrett, Julian Downward, Elza C. de Bruin

**Affiliations:** 1 Translational Medicine, Oncology, AstraZeneca, Cambridge, CB4 0WG UK; 2 Bioscience, Oncology, AstraZeneca, Cambridge, CB2 0RE UK; 3 Precision Medicine and Biosamples, BioPharmaceutical, AstraZeneca, Cambridge, CB4 0WG UK; 4 Translational Medicine, Oncology, AstraZeneca, Waltham, MA 02451 USA; 5 Oncogene Biology, Francis Crick Institute, London, NW1 1AT UK

**Keywords:** Oncogenes, Cancer, Lung cancer

## Abstract

The development of covalent inhibitors against KRAS G12C represents a major milestone in treatment of RAS-driven cancers, especially in non-small cell lung cancer (NSCLC), where KRAS G12C is one of the most common oncogenic driver. Here we investigated if additional KRAS mutations co-occur with KRAS G12C (c.34G>T) in NSCLC tumours and if such mutation co-occurrence affects cellular response to G12C-specific inhibitors. Analysis of a large cohort of NSCLC patients whose tumours harboured KRAS mutations revealed co-occurring *KRAS* mutations in up to 8% of tumours with the *KRAS* c.34G>T mutation. *KRAS* c.35G>T was the most frequently co-occurring mutation, and could occur on the same allele (in cis) translating to a single mutant KRAS G12F protein, or on the other allele (in trans), translating to separate G12C and G12V mutant proteins. Introducing *KRAS* c.35G>T in trans in the KRAS G12C lung cancer model NCI-H358, as well as the co-occurrence in cis in the KRAS G12F lung cancer model NCI-H2291 led to cellular resistance to the G12C-specific inhibitor AZ’8037 due to continuing active MAPK and PI3K cascades in the presence of the inhibitor. Overall, our study provides a comprehensive assessment of co-occurring *KRAS* mutations in NSCLC and in vitro evidence of the negative impact of co-occurring *KRAS* mutations on cellular response to G12C inhibitors, highlighting the need for a comprehensive *KRAS* tumour genotyping for optimal patient selection for treatment with a KRAS G12C inhibitor.

## Introduction

Activating mutations in the Kirsten rat sarcoma viral oncogene homolog (*KRAS*) gene are one of the most common oncogenic driver mutations in human cancers, including non-small cell lung cancer (NSCLC), where they account for about 30% of lung adenocarcinomas in western countries and 10% in Asian countries^1^. Up to 89% *KRAS* driver mutations occur in codon 12, whilst mutations in codons 13 and 61 represent the majority of the remaining *KRAS* oncogenic mutations^2,3^. The two most common KRAS mutations in NSCLC are G12C (~ 40%) and G12V (~ 22%), leading to an impairment of KRAS intrinsic hydrolytic activity and increased activation of KRAS oncoprotein^3^.

Despite being a well-known oncogenic driver for several decades^4^, KRAS has proven a challenging therapeutic target^5^. However, recently, several covalent inhibitors against KRAS G12C have been developed and show promising preclinical^6–9^ and early clinical efficacy data in KRAS G12C mutant lung cancers^9–12^. These compounds function by their specific and irreversible binding to the mutant cysteine, thereby trapping KRAS G12C in an inactive GDP-bound state^13^. Sotorasib (Lumakras, AMG510) has been granted accelerated approval by FDA in May 2021^14^, several G12C-specific inhibitors are currently evaluated in clinical trials and additional compounds are under development and expected to enter clinical trials in a near future^15^. Taking into account that KRAS G12C occurs in numerous cancer types, such as lung, colorectal, and cervical adenocarcinomas^16,17^, this class of agents brings a unique therapeutic opportunity to treat KRAS G12C-driven cancers.

Some evidence suggests that additional *KRAS* mutations can co-occur with KRAS G12C, and has been observed in colorectal, gastric and lung cancer patients^18–23^. These co-occurring *KRAS* mutations can be detected either in different tumours lesions in the same patient^20^, in distinct clones within the same tumour^19^ or even in the same tumour cell^22,23^. For instance Macedo et al. reported that 2.1% of all *KRAS* mutant tumours across numerous colorectal cancer studies harboured multiple *KRAS* mutations^18^. Interestingly, a multi-region whole-exome sequencing approach on 100 early-stage NSCLC tumours revealed clonal occurrence of KRAS G12C together with a subclonal KRAS G12V in one patient^23^. Considering that the G12C-specific inhibitor only targets the G12C amino acid change, we hypothesised that patients whose tumours harbour a co-occurring *KRAS* mutation may not respond well to this type of agents.

To determine the frequency of co-occurring *KRAS* mutations in NSCLC and potential impact on the response to KRAS G12C-specific inhibitors, we assessed the *KRAS* mutation status in a large cohort of locally advanced or metastatic NSCLC samples (n = 2306), assayed for enrolment into the SELECT-1 phase 3 trial (NCT01933932), and examined how some of these co-occurring mutations influence response of lung cancer in vitro models to the KRAS G12C-specific inhibitor AZ’8037 (also known as “compound 25”)^24^. Our results show that the *KRAS* c.35G>T mutation is most frequently co-occurring with the *KRAS* c.34G>T (G12C) mutation in *KRAS*-mutant NSCLC patients, co-occurring either in cis (i.e. on the same allele, and leading to a G12F mutation) or in trans (i.e. on different alleles, and translating to a G12C & G12V). Both scenarios impair sensitivity to AZ’8037 in vitro. Our results therefore highlight the importance of comprehensive *KRAS* genotyping in addition to the c.34G>T mutation to identify patients most likely to benefit from KRAS G12C-specific inhibitors.

## Results

### KRAS G12C can co-occur with other activating KRAS mutations in NSCLC

In order to assess the prevalence of co-occurring *KRAS* mutations in NSCLC patients, we retrospectively analysed *KRAS* genotypes from a large cohort of patients screened for enrolment in the SELECT-1 Phase 3 study (NCT01933932), where mutant *KRAS* was a key eligibility criteria^25^. In total, 2306 tumour samples were sequenced and actionable *KRAS* mutations were detected in 1084 samples (47%) (Fig. S1A). As expected, the vast majority of mutations were identified in codons 12, 13 and 61 and the most frequently detected *KRAS* mutation was G12C (c.34G>T), being found in 429 samples (40%) as a single *KRAS* mutation (Fig. S1B). A full list of oncogenic *KRAS* mutations and their frequencies is shown in Table S1.

**Figure 1:**
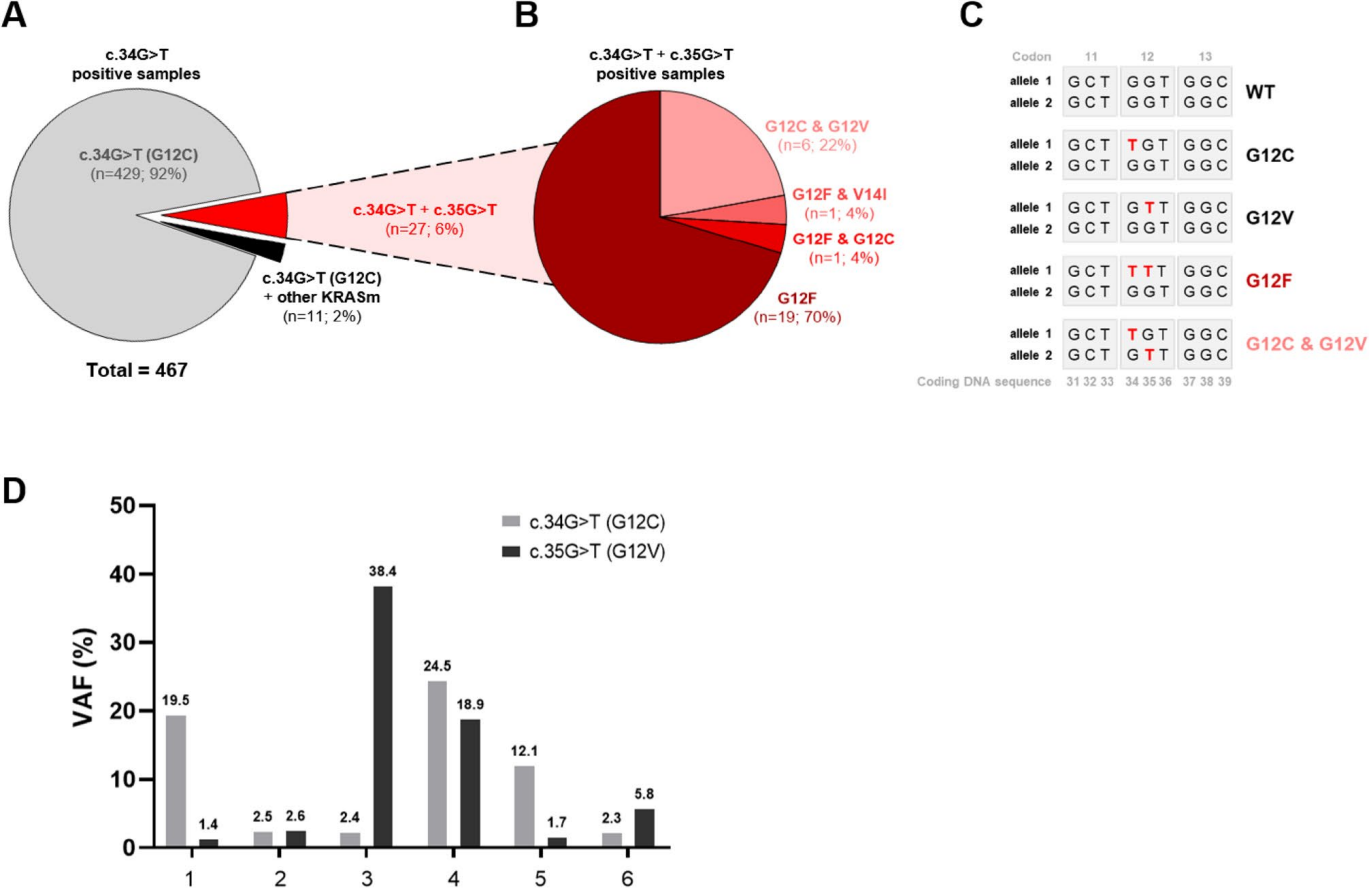
KRAS G12C (c.34G>T) co-occurrence with other activating *KRAS* mutations in NSCLC patients. (**A**) A pie chart of *KRAS* mutations in 467 G12C-positive samples. 27 samples showed co-occurrence of *KRAS* c.34G>T with c.35G>T. 11 samples were positive for KRAS c.34G>T and activating mutation in another codon (codon 13 or 14). (**B**) Frequency of individual *KRAS* genotypes in 26 samples with *KRAS* c.34G>T and c.35G>T co-occurring mutations. (**C**) A simplified scheme of two *KRAS* alleles and the effect of c.34G>T and c.35G>T co-occurrence on *KRAS* genotype. Co-occurrence in cis translates to KRAS G12F, whereas in trans produces both KRAS G12C and G12V proteins. (**D**) KRAS G12C and G12V VAFs in the six tissue samples where these mutations co-occurred in trans.

Interestingly, in addition to being identified as a single *KRAS* mutation, the c.34G>T mutations co-occurred with another *KRAS* mutation(s) in 37 samples, 8% of all c.34G>T mutation-positive cases (Fig. 1A, Table S1). The most frequently co-occurring mutation was *KRAS* c.35G>T, which was detected in 27 samples (6%). Co-occurring c.34G>T and c.35G>T mutations were present either in cis, leading to the amino acid change G12F (21 cases) (Fig. 1B,C; Table S1), or in trans, leading to two mutant KRAS proteins: KRAS G12C and KRAS G12V (6 patients) (Fig. 1B,C; Table S1). Importantly, these co-occurring *KRAS* mutations were also detected in other independent publicly available datasets at comparable frequencies (Table 1)^26–28^.

**Table 1:** Frequency of *KRAS* c.34G>T (G12C) alone or co-occurring with another actionable *KRAS* mutation in four independent datasets. KRASm (KRAS non-synonymous mutations).

KRAS mutation	Our study (2306 patients; 1084 KRASm patients)		TCGA Nat Genet 2016; LUAD (660 patients; 214 KRASm patients)^a^		TCGA PanCancer Atlas; LUAD (566 patients; 168 KRASm patients)^b^		MSKCC Cancer Discov 2017; metastatic LUAD (860 patients; 236 KRASm patients)^c^	
	# patients	% patients	# patients	% patients	# patients	% patients	# patients	% patients
G12C	429	91.9	96	98.0	68	97.1	101	97.1
G12F	19	4.1	2	2.0	0	0.0	1	1.0
G12C & V14I	10	2.1	0	0.0	0	0.0	0	0.0
G12C & G12V	6	1.3	0	0.0	2	2.9	1	1.0
G12F & G12C	1	0.2	0	0.0	0	0.0	1	1.0
G12F & V14I	1	0.2	0	0.0	0	0.0	0	0.0
G12C & G13D	1	0.2	0	0.0	0	0.0	0	0.0
Total	467	100	98	100	70	100	104	100

^a^Campbell et al.^28^.

^b^Hoadley et al.^27^.

^c^Jordan et al.^26^.

As a clonal KRAS G12C and subclonal KRAS G12V have been previously described in one early-stage NSCLC tumour in the TRACERx study^23^, we next aimed to assess possible evolution of the mutational events in the six G12C & G12V double-positive cases by investigating the G12C and G12V variant allele frequencies (VAFs). Interestingly, we did not observe any trend of one KRAS mutation being more likely detected at higher VAF over the other (Fig. 1D), indicating that either mutation could be evolutionally older.

In the majority of samples with c.34G>T and c.35G>T co-occurring in cis and translating to G12F, both mutations were identified with similar VAFs, suggesting that a single event led to the introduction of two nucleotide changes simultaneously. Interestingly, one sample harboured both KRAS G12C (c.34G>T; VAF = 2.6%) and G12F (c.34_35GG>TT; VAF = 38.4%) mutations (Fig. S2C), and it is therefore possible that G12C could be evolutionary older and could become G12F after a second independent hit to the same allele.

### KRAS G12C & G12V double mutant and KRAS G12F cell line models are resistant to the G12C-specific inhibitor AZ’8037 in vitro

**Figure 2:**
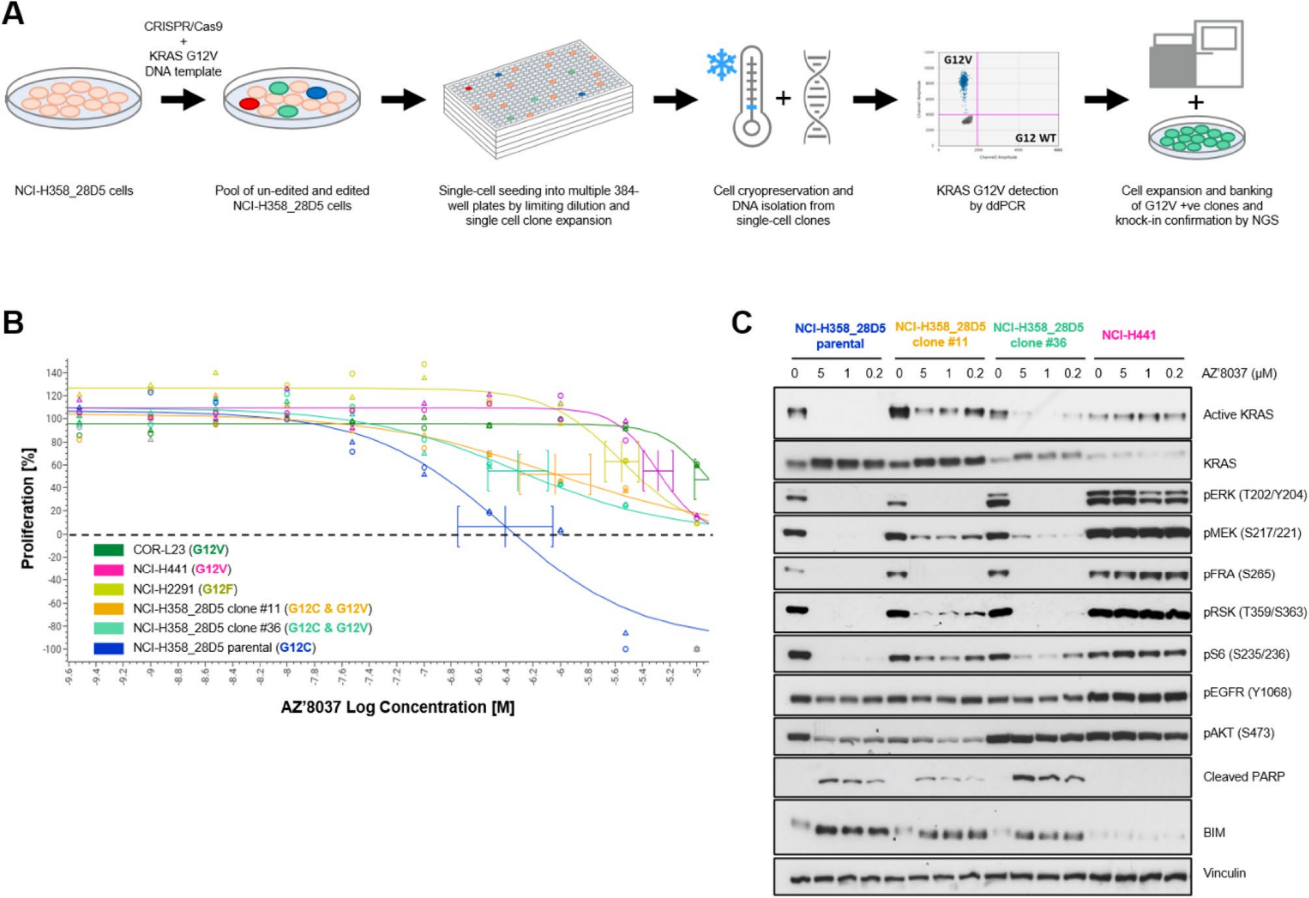
In vitro functional analysis of the KRAS G12C & G12V model. (**A**) Experimental design of CRISPR-based knock-in (KI) of *KRAS* G12V in NCI-H358_28D5 cells and clone validation by KRAS genotyping (details in the Materials and Methods section). (**B**) Effect of AZ’8037 treatment on the viability of *KRAS* mutant cell line models grown in 2D monolayer. Representative data from three independent experiments is shown, error bars represent mean ± SD from replicate wells. The dashed line represents cell count at Day 0. The curve bottom below Day 0 level indicates induction of cell death after 5 days of treatment, while above 0 indicates cytostatic effect. (**C**) Effect of AZ’8037 on active KRAS level and downstream signalling. Immunoblot analysis of cellular extract from *KRAS* mutant cell line models grown in 2D monolayer and treated with vehicle (DMSO; marked as 0) or the indicated concentrations of AZ’8037 for 16 h. Full-length blots included in a Supplementary Information file (Fig. S4).

We next determined whether the co-occurrence of *KRAS* c.34G>T with c.35G>T, either in cis (translating to G12F) or in trans (translating to G12C and G12V double mutant) impacts sensitivity of NSCLC cancer cell lines to the G12C-specific inhibitor AZ’8037 (also known as “compound 25”)^24^ in vitro. We used the NCI-H2291 cell line to assess the response of G12F-mutant NSCLC cells to the G12C-specific inhibitor, and employed CRISPR/Cas9 technology to knock-in *KRAS* c.35G>T into the NCI-H358_28D5 NSCLC cell line (Fig. 2A), a heterozygous KRAS G12C cell line carrying four G12C and two WT alleles (Fig. S3A). CRISPR-induced knock-in efficiency was 8% with five clones harbouring the KRAS G12V mutation (Fig. S3B). Amplicon sequencing of four of the KRAS G12V-positive clones revealed that clones #11 and #36 harboured G12C and G12V in trans and no additional alteration of the locus in other alleles (Fig. S3C,D), and thus these two clones have been selected for further in vitro functional analysis.

We then assessed the sensitivity of NCI-H2291 cells (G12F) and clones #11 and #36 (G12C and G12V NCI-H358_28D5 double mutants) to AZ’8037 and compared them to the parental NCI-H358_28D5 cells (G12C), used as a positive control, and NCI-H441 and COR-L23 (both G12V), which were used as negative controls. Treatment with AZ’8037 inhibited the proliferation of the parental NCI-H358_28D5 cells (GI_50_ 0.11 μM) and induced cell death at higher concentrations (Fig. 2B, Table S2). In contrast the G12V (NCI-H441 & COR-L23) and G12F (NCI-H2291) cells were relatively insensitive to compound treatment with GI_50s_ above 3.2 μM, in line with the fact that these cell line do not harbour a G12C mutation, which is targeted by the compound (Fig. 2B, Table S2). Interestingly, both single cell clones harbouring the G12C & G12V double mutations showed decreased sensitivity to AZ’8037 with higher GI_50_ when compared to the parental NCI-H358_28D5 cells (Fig. 2B, Table S2).

Moreover, AZ’8037 only had a cytostatic effect in these cell lines with no induction of cell death. The decreased sensitivity of the G12C & G12V double mutant clones to G12C inhibition is likely to be mediated by incomplete inhibition of KRAS activity and KRAS effector pathway signalling driven by the KRAS G12V mutant allele as shown by only a partial reduction in the levels of pS6 and pMEK and a modest induction of the pro-apoptotic biomarker BIM (Fig. 2C, Fig. S4).

## Discussion

In this analysis of a large cohort of NSCLC patients with *KRAS* mutant tumours, we observed co-occurring *KRAS* mutations in 8% of the *KRAS* c.34G>T mutant NSCLC tumours, of which *KRAS* c.35G>T was most frequently detected. *KRAS* c.35G>T can co-exist with KRAS c.34G>T either in cis or in trans, each leading to distinct protein changes. When present in cis, i.e. on the same allele, the mutations translate to KRAS G12F protein, whereas in trans, the mutations lead to two KRAS mutant proteins, KRAS G12C and KRAS G12V. Importantly, CRISPR-induced knock-in of the c.35G>T mutation in trans (G12V) in the KRAS G12C lung cancer model NCI-H358_28D5, as well as the co-occurrence in cis in the KRAS G12F lung cancer model NCI-H2291, showed a strongly decreased sensitivity to the G12C-specific inhibitor AZ’8037 in vitro compared to the KRAS G12C lung cancer cell line.

Although we focused our functional validation on the most abundant concomitant mutation, the c.35G>T, co-mutations in other *KRAS* codons (Table 1), in particular the activating mutation p.V14I^29^, could potentially also compromise the inhibitory effect of G12C inhibitors. According to World Health Organization (WHO) approximately 2.1 million new lung cancer cases were diagnosed globally in 2018^30^. As NSCLC cases account for 85% of lung cancers, of which approximately third are *KRAS* mutant lung adenocarcinomas, with 40% tumours harbouring KRAS G12C^31^, thousands of newly diagnosed KRAS G12C mutant tumour each year would be expected to harbour another concomitant *KRAS* mutation which could affect patients’ response to G12C inhibitors.

To our knowledge, we provide the largest and most comprehensive assessment of multiple *KRAS* mutations in NSCLC to date, which allowed for an accurate estimation of the frequency of these events. Our data builds on the limited information available from few smaller studies about the prevalence and biology of co-occurring *KRAS* mutations in colorectal, gastric and lung cancer patients^18–23^, where multiple *KRAS* mutations were detected either in different tumour lesions in the same patient^20^, distinct cell clones within the same tumour^19^ or in the same tumour cell^22,23^. In the TRACERx study, multi-region whole-exome sequencing on 100 early-stage NSCLC tumours revealed 7 KRAS G12C-positive tumours, with one tumour harbouring co-occurring KRAS G12C and G12V mutations. Interestingly, the KRAS G12C mutation was clonal, whereas the KRAS G12V was observed at subclonal level, indicating KRAS codon 12 can be hit twice in the same cell during the course of NSCLC development^23^. The KRAS protein change then depends on which allele is hit the second time. One patient from our study harboured both G12F and G12C mutations and it is therefore possible that an originally G12C tumour could become G12F after a second hit to an already mutant allele and the cells with and without the second hit continued to expand alongside. This observation of a second hit leading to G12F suggests that such second hit may be one of the mechanisms that tumours could potentially employ to acquire resistance to KRAS G12C-specific inhibitors. Recent data from limited number of KRAS G12C mutant cancer patients indeed show accumulation of secondary KRAS mutations in codons 12, 13, 61, 68, 95 and 96 upon treatment with G12C inhibitor adagrasib (MRTX849)^32,33^. Interestingly, recent in vitro study showed that KRAS R68S and Y96C confer resistance to both adagrasib and sotorasib, however the H95D, H95Q, or H95R adagrasib-resistance mutations do not confer in vitro resistance to sotorasib, suggesting differences between KRAS G12C inhibitors^34^. Further analysis in clinical samples collected upon progression on a KRAS G12C inhibitor is warranted to assess this hypothesis.

We however cannot rule out the possibility that, in our study, the two KRAS mutations detected in six G12C & G12V and one G12C & G12F cases arose in separate cell clones. Nevertheless, even if this was the case, the results from our in vitro study suggest that the G12C-positive clone would be expected to be diminished by AZ’8037, whereas the G12V or G12F clones would not respond to the G12C inhibitor, possibly leading to tumour progression. Thus, our results indicate that the detection of both G12C and G12V mutants in a NSCLC tumour could lead to a reduced sensitivity of the tumour to G12C inhibitor when compared to G12C-positive tumours.

We were able to detect both G12C & G12V and G12F mutants in publicly available datasets at comparable frequencies to our study (Table 1)^26–28^, indicating that the KRAS G12C mutation co-occurrence is a consistent finding across lung cancer studies and patient populations. In fact, the frequency of KRAS double mutants is likely underestimated as some subclonal *KRAS* mutants could have been missed if multi-region tumour sequencing is not performed^23^.

Importantly, the prevalence of KRAS G12F in our study is four-times higher than the prevalence of the G12C & G12V double mutation. As AZ’8037 was designed to bind to the mutant Cys12 residue it should not inhibit G12F protein, which was confirmed by the resistance of G12F-mutant NCI-H2291 cells to AZ’8037. Therefore, no clinical benefit of G12F tumours to G12C inhibitors would be expected. Thus, our results highlight the importance of a comprehensive *KRAS* hotspot genotyping for patient selection for treatment with G12C inhibitors, especially to discriminate G12C-positive case from G12C & G12V or G12F cases. Numerous *KRAS* mutation detection technologies are currently available for sample genotyping, including qPCR-, ddPCR-, NGS- and mass spectrometry-based methodologies^35^. A comprehensive comparison of different approaches by Sherwood et al. highlights the advantages and disadvantages of each method. Validated qPCR-based tests for use in clinical samples such as the Therascreen KRAS RGQ PCR Kit or the Cobas® KRAS Mutation Test have the fastest turnaround time, but only detect and identify mutations in codons 12/13 or do not discriminate between mutations at the amino acid level for codons 12/13/61, respectively^35,36^. Importantly, neither of the two assays detect G12F or report multiple mutations, and thus these assays are not suitable for identification of G12C & G12V or G12F cases. On the other hand, NGS assays, despite requiring more tissue and a longer turnaround time, are able to precisely genotype both single and co-occurring KRAS mutations even at low variant allele frequencies ^35^ and might be a preferred option for detection of co-occurring KRAS mutations.

In summary, our results show that co-occurring *KRAS* mutations exist in up to 8% of *KRAS* c.34G>T-positive NSCLC tumours, with *KRAS* c.35G>T being the most frequent and translating to either G12F or G12C & G12V double mutant, when occurring in cis or trans respectively. The G12C inhibitor AZ’8037 has a cytotoxic effect in G12C-mutant lung cancer cells, but G12C & G12V or G12F cell line models showed resistance to the treatment in vitro. Overall, our study provides a novel in vitro evidence of the impact of multiple *KRAS* mutations on cellular response to G12C inhibitors and highlights the importance of a comprehensive *KRAS* hotspot genotyping for patients treated with KRAS G12C inhibitors.

## Materials and methods

### Patients

We retrospectively evaluated genomic data from tumour biopsies collected from NSCLC patients screened for enrolment into the SELECT-1 study (NCT01933932). Full details of the methodology of the SELECT-1 study have been published previously^25,37^. All methods were carried out in accordance with relevant guidelines and regulations. The study was performed in accordance with the ethical principles of the Declaration of Helsinki and the International Conference on Harmonisation of Technical Requirements for Registration of Pharmaceuticals for Human Use Good Clinical Practice guidelines. The trial protocol states ‘*An Institutional Review Board (IRB)/Ethics Committee should approve the final study protocol, including the final version of the Informed Consent Form and any other written information and/or materials to be provided to the patients. The investigator will ensure the distribution of these documents to the applicable IRB/Ethics Committee, and to the study site staff.*’ and the protocol was approved by an IRB/Ethics Committee at each participating site. All patients provided written informed consent before any study-specific procedures, sampling, and analyses from an early phase clinical study as detailed in the clinical study protocol (NCT01933932).

### Tumour DNA extraction and KRAS genotyping

Tumour DNA was isolated using the cobas® DNA Sample Preparation Kit (Roche) as per manufacturer’s instructions and KRAS amplicon sequencing was outsourced to SeqWright (Houston, Texas, USA) and conducted on Illumina instruments. Only mutations with 15 + entries in lung tissue in the COSMIC database (https://cancer.sanger.ac.uk/cosmic) and/or classified as pathogenic or likely pathogenic in ClinVar (https://www.ncbi.nlm.nih.gov/clinvar/) were considered as hotspot mutations. Mutations reported less than 15-times in lung tissue in COSMIC and/or not recognised by the ClinVar database were considered as not actionable KRAS mutations.

### Cell lines and reagents

The NCI-H358 (KRAS G12C) cell line was obtained, authenticated, and cultured as recommended by the American Type Culture Collection (ATCC). NCI-H358-ODIN-Cas9-T2A-GFP cells were engineered as previously described^38^. Briefly, doxycycline inducible Cas9 nuclease was inserted into the AAVS promoter using Zink Fingers. Single-cell clones were prepared using limiting dilution of the NCI-H358-ODIN-Cas9-T2A-GFP cell pool and expanded for several weeks. Clone NCI-H358-ODIN-Cas9-T2A-GFP_28D5 (referred to as NCI-H358_28D5 in this manuscript) was used for further CRISPR experiments. NCI-H441 and NCI-H2291 were obtained from ATCC and COR-L23 from the European Collection of Authenticated Cell Cultures (ECACC). Cells were cultured in RPMI 1640 medium (Gibco) supplemented with 10% FCS (Gibco) and 2 mM Glutamine (ThermoFisher) at 37 °C in a humidified atmosphere with 5% CO2. All cell lines were confirmed to be negative for mycoplasma. Any other reagents were purchased from Sigma unless mentioned otherwise.

### CRISPR-based knock-in (KI) of KRAS G12V into the NCI-H358_28D5 cell model

CRISPR/Cas9 technology has been used in order to knock-in (KI) KRAS G12V mutation into the NCI-H358_28D5 cellular model. Briefly, NCI-H358_28D5 cells were treated with 100 ng/mL Doxycycline (Sigma) for 24 h to induce Cas9 expression, following by electroporation at 1000 V, 40 ms, 1 pulses (Neon Transfection System, ThermoFisher Scientific) with Alt-R® CRISPR-Cas9 tracrRNA and Alt-R® CRISPR-Cas9 sgRNA (Integrated DNA Technologies) with sequence 5′-CTTGTGGTAGTTGGAGCTGGTGG-3′ in conjunction with a synthetic single-strand DNA oligo donor (Ultramer oligo, Integrated DNA Technologies) with homology arms to the WT KRAS allele and the following sequence: ATGCATATTAAAACAAGATTTACCTCTATTGTTGGATCATATTCGTCCACAAAATGATTCTGAATTAGCTGTATCGTCAAGGCACTCTTGCCTACGCCAACTGCTCCAACTACCACAAGTTTATATTCAGTCATTTTCAGCAGGCCTTATAATAAAAATAATGAAAATGTGACTATATTAGAACATGTCACACATAAGGT. The sequence contains KRAS G12V (c.35G>T) together with a silent “blocking” mutation (c.33T>A) in order to block re-cutting of the locus and increased homology-directed repair (HDR) accuracy^39^. 24 h post-transfection, the cells were seeded in multiple clear bottom 384-well plates (Sartorius) at concentration < 1 cell/well, single-cell clones were clonally expanded for several weeks and a fraction of each clone was used for crude DNA lysis for further DNA genotyping by ddPCR. Another fraction of cells was cryopreserved in FCS (Gibco) supplemented with 10% DMSO (Sigma) and only selected clones were further expanded and used in in vitro experiments.

### Genotyping of selected CRISPR clones by ddPCR

DNA from the parental NCI-H358_28D5 cells was extracted using the DNeasy Blood & Tissue Kit (Qiagen) according to manufacturer’s instructions and in total 62 single cell clones were subjected to crude DNA lysis as described previously^40^. Reaction volumes were made up to 20 μl and partitioned to up to 20,000 droplets using a ddPCR Auto Droplet Generator (Bio-Rad). For mutation analysis the following conditions were used: 95 °C for 10 min followed by 40 cycles of 94 °C for 30 s then 60 °C for 60 s, ramp rate 2 °C/second, and final incubation 98 °C for 10 min. The subsequent analysis was done on a Bio-Rad QX200 droplet reader, and analysed using QuantaSoft Analysis Pro software v1.0.596 (Bio-Rad). Primer and probe sequences are listed in Table S3. CRISPR-induced knock-in efficiency was 8% with five clones harbouring the KRAS G12V mutation (Fig. S3B).

### Next-generation sequencing of a KRAS locus and bioinformatics

Genomic DNA was isolated from four CRISPR clones (#11, #13, #34, #36) using the DNeasy Blood & Tissue Kit (Qiagen) according to manufacturer’s instructions. 12.5 ng of genomic DNA were amplified using a two-step PCR that added unique library bar-codes, heterogeneity spacers and Illumina MiSeq adapters as described previously^41^. Primer sequences for two-step PCR are attached in Table S4. Samples were sequenced using a MiSeq® Reagent Nano Kit v2 (500 Cycles) (Illumina) on a MiSeq instrument (Illumina). Quantification and classification of the sequences was done using the following tools: Fast Length Adjustment of Short reads (FLASH v1.2.11) was used to group paired reads. BWA-MEM was used to align to the human genome (hg19) or the BFP coding sequence. Samtools was used to generate sorted, indexed BAM files. Samtools was used to generate data for variant calling with the following options: minimum read depth 1000, minimum quality 25, minimum allele frequency 0.01 (1%), maximum mismatch 100, and trim 20. Amplicon sequencing mapping summary is presented in Table S5. Clones #11 and #36, which harboured G12C and G12V in cis and no additional alteration of the locus in other alleles (Fig. S3C,D), were selected for further in vitro functional analysis.

### Cell proliferation assay

All cells were cultured in RPMI-1640 containing 10% Foetal Calf Serum (FCS) and 2 mM l-Glutamine. Proliferation was assessed by seeding cells into 384-well clear bottom plates (Greiner) in 70 µL of RPMI-1640 growth media at 250–1000 cells/well. Plates were incubated for 24 h at 37 °C, 5% CO_2_ and either processed immediately (day 0) or treated with a dose range of AZ’8037 prepared in DMSO using an ECHO 555 liquid handler (Labcyte Inc.) and incubated for a further 5 days. The number of dead and live cells at day 0 and day 5 were determined using a sytox green assay. In brief, 2 µM sytox green nucleic acid dye (Life Technologies; in TBS + 5 mM EDTA) was added (5 µL/well) and plates incubated for 1 h at 37 °C. The number of green cells in each well (dead cells) was measured using an Acumen Explorer high-throughput cell imager (TTP Labtech Ltd.) using laser voltage set at 425 V. Next, 0.25% w/v Saponin (Sigma; in TBS + 5 mM EDTA) was added (10 µL/well) overnight at room temperature to permeabilise the cells before re-counting the number of green cells and therefore allowing a total cell count. Number of live cells were calculated by subtracting the dead cell count from the total cell count.

### Ras activity assay

400,000 cells were plated in each well of 6 well tissue culture plate. After 24 h plates were dosed with three dilutions of AZ’8037 (5, 1 and 0.2 µM) along with DMSO control. After 16 h post-dosing, cells were washed in ice-cold PBS + 2 mM MgCl_2_ and then lysed using Ras activity buffer provided in the Active Ras pull down and detection kit (Thermo Fisher). Cell lysates were centrifuged at 13 k RPM for 10 min at 4 °C. The supernatant was collected and protein quantified using DC protein quantification assay. 100 µg protein was added in 200 µL Ras activity buffer and 50 µl of Glutathione Sepharose bead and mixed for 2 h at 4 °C. Beads were washed three times with Ras activity buffer and protein was eluted with 2 × SDS loading buffer. The pull down samples and the input samples were analysed in NuPAGE SDS PAGE and transferred using iBLOT. Membranes were incubated with primary antibodies at 4 °C overnight, washed three times with TBST (TBS + 0.5% Tween 20), incubated with secondary antibody for 2 h at room temperature, washed three times with TBST and developed using SuperSignal™ West Dura substrate (Thermofisher) and visualised using Gbox. Details of the primary and secondary antibodies listed in Table 2.

**Table 2:** List of primary and secondary antibodies used in the Ras activity assay.

Antibody	Species	Source	Catalogue no.	Dilution
KRas	Mouse	LS Bio	C175665	1:2000
pMEK S217/221	Rabbit	Cell signalling	9154	1:1000
pERK T202/Y204	Mouse	Cell signalling	9106	1:1000
pAKT (S473)	Rabbit	Cell signalling	4060	1:1000
BIM	Rabbit	Cell signalling	2933	1:1000
Vinculin	Mouse	Sigma	V9131	1:10,000
pFRA (S265)	Rabbit	Cell signalling	3880	1:1000
p-EGFR (Y1068)	Rabbit	Cell signalling	3777	1:1000
Cleaved PARP	Rabbit	Cell signalling	9541	1:1000
pRSK T359/S363	Rabbit	Cell signalling	9344	1:1000
Phospho-S6 Ribosomal Protein (S235/236)	Rabbit	Cell signalling	4858	1:2000
Anti-rabbit IgG HRP-linked	Goat	Cell signalling	7074	1:5000
Anti-mouse IgG HRP-linked	Goat	Cell signalling	7076	1:5000

### Statistics

The graphs, descriptive statistics and statistical tests were made using the GraphPad Prism software (version 8.0.1).

## Supplementary Information

Supplementary Information.
