# Supplementary material for: Concomitant KRAS mutations attenuate sensitivity of non-small cell lung cancer cells to KRAS G12C inhibition
Source: Sci Rep. 2022 Feb 17;12:2699. doi: 10.1038/s41598-022-06369-3 (PMC8854729; doi:10.1038/s41598-022-06369-3)
Supplement: Supplementary file 1 — Supplementary Information. [file 41598_2022_6369_MOESM1_ESM.pdf]

Supplementary information

Concomitant *KRAS* mutations attenuate sensitivity of non-small cell lung cancer cells to KRAS G12C inhibition

Tereza Vaclova[1], Atanu Chakraborty[2], James Sherwood[3], Sarah Ross[2], Danielle Carroll[1], J. Carl Barrett[4], Julian Downward[5], Elza C. de Bruin[1]*

[1]Translational Medicine, Oncology, AstraZeneca, Cambridge CB4 0WG, UK.

[2]Bioscience, Oncology, AstraZeneca, Cambridge CB2 0RE, UK.

[3]Precision Medicine and Biosamples, BioPharmaceutical, AstraZeneca, Cambridge CB4 0WG, UK.

[4]Translational Medicine, Oncology, AstraZeneca, Waltham, MA 02451, USA.

[5]Oncogene Biology, Francis Crick Institute, London NW1 1AT, UK.

*To whom correspondence should be addressed: elza.de-bruin@astrazeneca.com

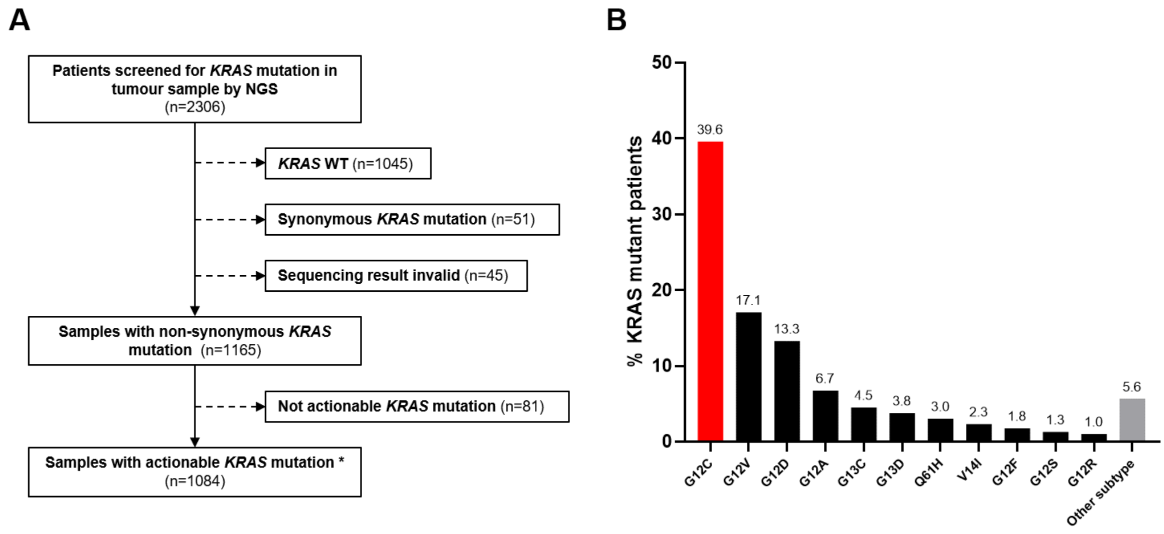

Fig S1: Tumour *KRAS* genotype analysis in patients screened for enrolment into the Select-1 trial. (A) From the 2306 screened patients, in total 1084 patients had detectable non-synonymous hotspot mutation in the *KRAS* gene. * Only mutations with 15+ entries in lung tissue in the COSMIC database (https://cancer.sanger.ac.uk/cosmic) and/or classified as pathogenic or likely pathogenic in ClinVar (https://www.ncbi.nlm.nih.gov/clinvar/) were considered as actionable. Mutations reported less than 15-times in lung tissue in COSMIC and not recognised by the ClinVar database were considered as not actionable *KRAS* mutations. (B) Histogram of a frequency of the main *KRAS* hotspot mutations in 1084 *KRAS* mutant tumour samples. Only mutations detected in ≥1% samples are specified. Only single KRAS mutants are shown in the counts. If more than one *KRAS* mutation/sample was detected the combination was considered as a separate subtype (details in **Table S1**). Individual multiple mutant subtypes were found in ≤1% samples and thus are included in the "other subtype" group.

Table S1: List of all actionable *KRAS* mutant subtypes detected in 1084 tumour samples. Samples with single or multiple *KRAS* mutations are specified.

KRAS protein change	# positive samples	% positive samples
G12C	429	39.6
G12V	185	17.1
G12D	144	13.3
G12A	73	6.7
G13C	49	4.5
G13D	41	3.8
Q61H	33	3.0
V14I	25	2.3
G12F	19	1.8
G12S	14	1.3
G12R	11	1.0
G12C & V14I	10	0.9
Q61L	9	0.8
G12C & G12V	6	0.6
G13V	4	0.4
Q61R	4	0.4
G12D & V14I	3	0.3
A59T	2	0.2
G12A & V14I	2	0.2
G12D & G13S	2	0.2
G12S & V14I	2	0.2
G12V & V14I	2	0.2
G13S	2	0.2
Q61K	2	0.2
A59T & V14I	1	0.1
G12A & G12R	1	0.1
G12A & G12V	1	0.1
G12F & V14I	1	0.1
G12F & G12C	1	0.1
G12C & G13D	1	0.1
G12D & G12V	1	0.1
G12D & G13C	1	0.1
G12D & V14I & Q61H	1	0.1
G12V & Q61H	1	0.1
G13C & G13V	1	0.1
Total	1084	100

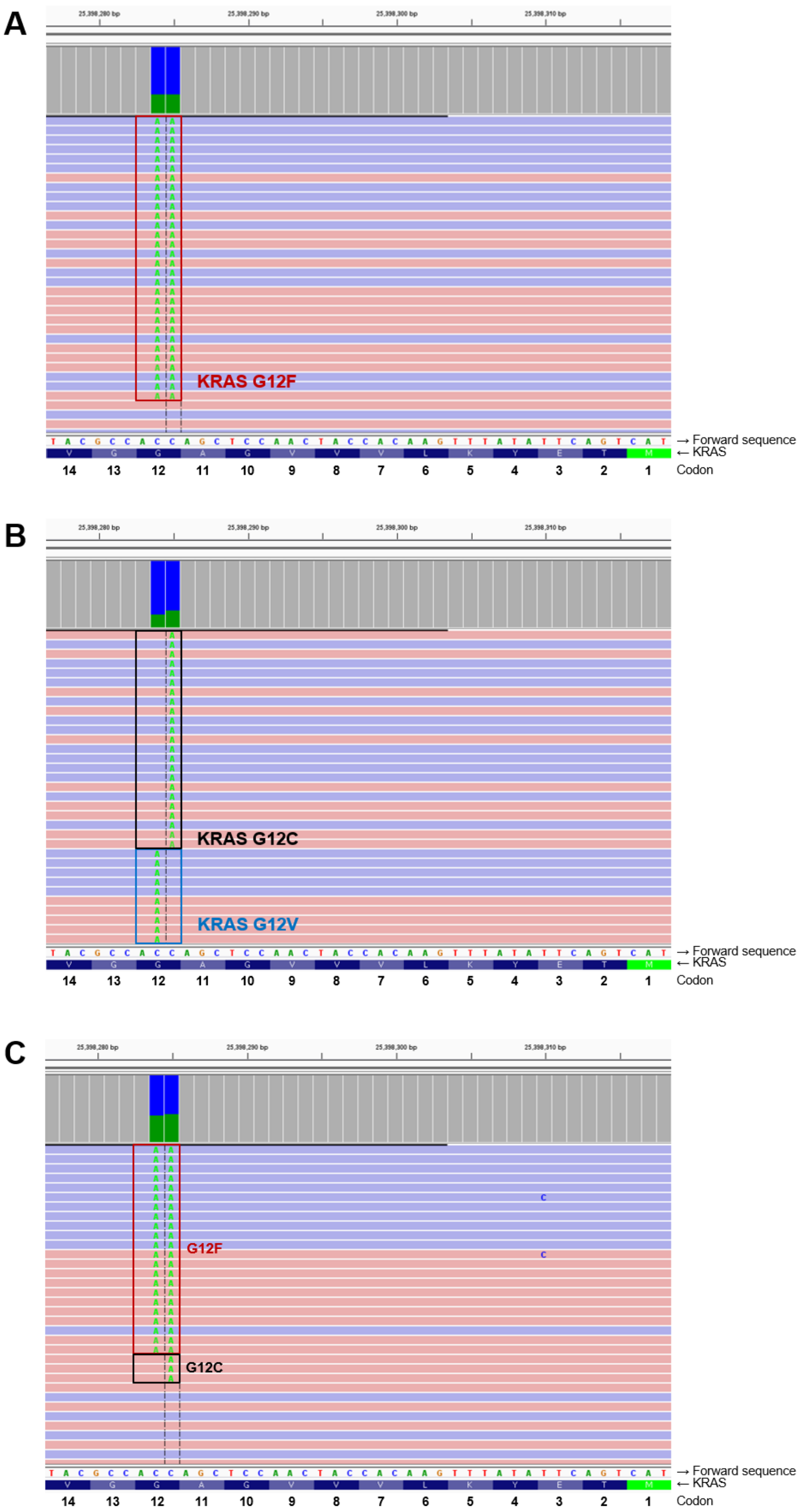
A
25,398,280 bp
25,398,290 bp
25,398,300 bp
25,398,310 bp
KRAS G12F
→ Forward sequence
← KRAS
14 13 12 11 10 9 8 7 6 5 4 3 2 1
Codon
B
25,398,280 bp
25,398,290 bp
25,398,300 bp
25,398,310 bp
KRAS G12C
KRAS G12V
→ Forward sequence
← KRAS
14 13 12 11 10 9 8 7 6 5 4 3 2 1
Codon
C
25,398,280 bp
25,398,290 bp
25,398,300 bp
25,398,310 bp
G12F
G12C
→ Forward sequence
← KRAS
14 13 12 11 10 9 8 7 6 5 4 3 2 1
Codon

Fig S2: Example sequencing results of c.34G>T and c.35G>T co-occurring mutations in *KRAS*. (A) Co-occurrence of c.34G>T and c.35G>T in cis, i.e. on the same sequencing read, results in the G12F amino acid change. (B) Co-occurrence of c.34G>T and c.35G>T in trans, i.e. on distinct sequencing reads, results in a translation to both G12C and G12V mutant proteins, which can be present in the same cell or in different cell clones within the tested tissue. (C) Co-occurrence of KRAS G12F and G12C mutations in 1 tumour sample. Number of sequencing reads was downsampled (as marked by the black line under the coverage track in A, B, C) for a better visualisation.

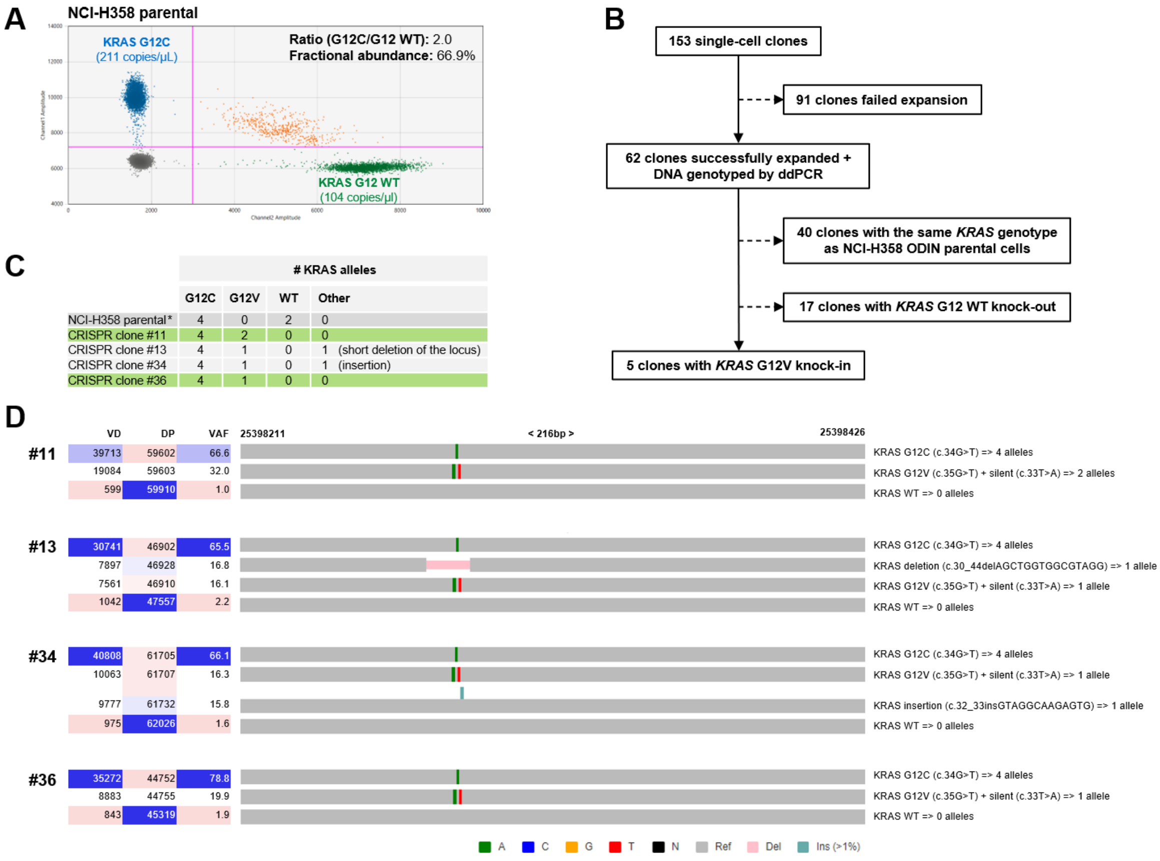

Fig S3: Establishment of NCI-H358_28D5 *KRAS* G12C+V double mutant cell line models. (A) *KRAS* codon 12 genotyping of the NCI-H358_28D5 parental cell line by ddPCR. Blue droplet population represents G12C-positive DNA molecules, green droplets mark the G12 WT DNA molecules, orange droplets carry both G12C and G12 WT DNA molecules. Ratio of G12C to G12 WT molecules is 2. (B) Individual CRISPR clone selection flowchart. In total 153 single-cell clones were isolated and cultured from the heterogeneous CRISPR cell pool. 62 clones (41%) were successfully expanded, cryopreserved and genotyped for KRAS G12V knock-in by ddPCR. 17 out of 62 genotyped clones (27%) exhibited knock-out of WT (wild type) KRAS G12 locus, which is an expected result as small insertions/deletions are a common feature of CRISPR knock-in experiments due to a high rate of an imprecise repair by the non-homologous end joining (NHEJ) pathway (1). (C) *KRAS* amplicon sequencing of four NCI-H358_28D5_G12V single cell clones, which were positive for G12V knock-in by a ddPCR assay. Clones #11 and #36 were both positive for the G12V knock-in and did not show any additional insertions/deletions, unlike clones #13 and #34. Green colour marks clones which were selected for future *in vitro* experiments. * *KRAS* genotyping the parental NCI-H358_28D5 cells was performed by ddPCR, as shown in A. (D) Frequency of individual alterations in the *KRAS* amplicon detected by NGS of four NCI-H358_28D5_G12V CRISPR cell clones. NGS confirmed the

presence of KRAS G12V in all four tested clones. KRAS G12V (c.35G>T) is accompanied by a silent mutation (c.33T>A) in order to prevent additional cleavage of knocked-in allele by the CRISPR/Cas9 machinery. In addition, small insertions/deletions were detected in clones #34 and #13, respectively, a common feature of CRISPR knock-in experiments due to a high rate of an imprecise repair by the non-homologous end joining (NHEJ) pathway. The amplicon length was 216bp. VD, variant depth; DP, read depth; VAF, variant allele frequency.

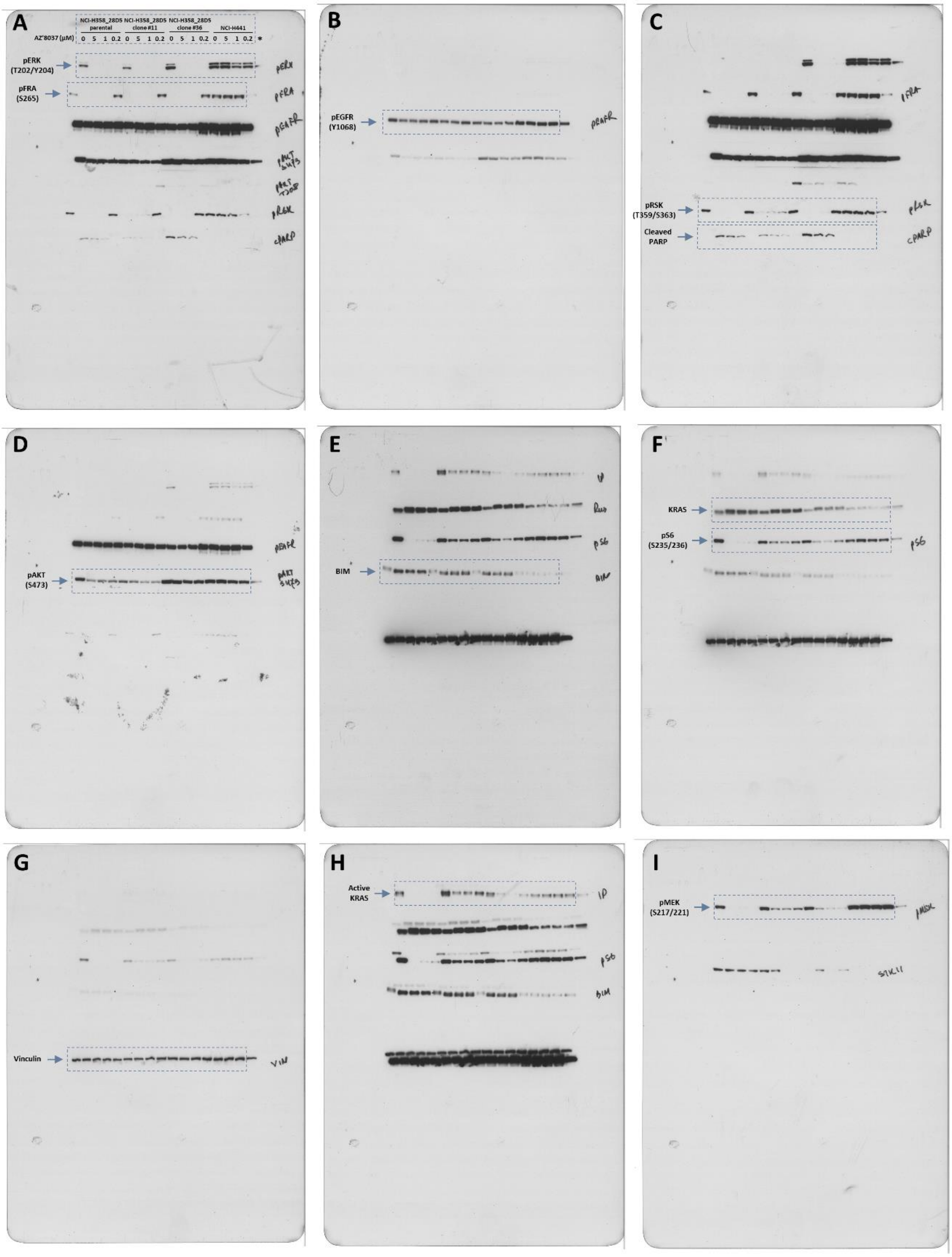

Fig S4: Full-length blots showing immunoblot analysis of cellular extract from *KRAS* mutant cell line models. Cells grown in 2D monolayer and treated with vehicle (DMSO; marked as 0) or the indicated concentrations of AZ'8037 for 16 hours. Blue dashed boxes show selection used for Fig 2C. No sample has been loaded in the last well marked with asterisk in the treatment conditions section in (A) due to previous observation of an insufficient transfer at that position. Individual scans show results for (A)

pERK (T202/Y204) and pFRA (S265), (B) pEGFR (Y1068), (C) pRSK (T359/S363) and cleaved PARP, (D) pAKT (S473), (E) BIM, (F) KRAS and pS6 (S235/236), (G) vinculin, (H) active KRAS, (I) pMEK (S217/221).

Table S2: Geometric mean of GI_{50} from replicate experiments for each cell line model. The error bars represent Geometric SD factor.

AZ'8037 GI_{50} (µM)	KRAS mutation status	Geometric Mean	Geometric SD Factor	Curve Bottom
NCI-H358_28D5 (parental)	G12C	0.11	1.28	-98
NCI-H358_28D5 clone #11	G12C & G12V	0.70	1.16	0
NCI-H358_28D5 clone #36	G12C & G12V	0.40	1.40	0
NCI-H2291	G12F	3.20	1.14	0
NCI-H441	G12V	5.51	1.06	0
COR-L23	G12V	10.00	1.00	0

Table S3: ddPCR primer and probe sequences.

PROBES/PRIMERS	Sequence	Match Tm (°C)	Mismatch Tm (°C)
WT Allele Probe	CC+A+C+CA+G+CTC	64.5	19.6
Mut Allele Probe	CC+A+A+CT+G+CT+CC	64.3	40.6
Forward Primer	AACCTTATGTGTGACATGTTCTAA	61.4	-
Reverse Primer	TGTATCGTCAAGGCACTCTT	61.5	-

Table S4: Primers for amplicon sequencing. Adapter sequences for binding indexing primers are in lower case letters.

PRIMERS	Sequence
Forward primer	tcgtcggcagcgtcagatgtgtataagagacagTGTATTAAAAGGTACTGGTGGAGT
Reverse primer	gtctcgtgggctcggagatgtgtataagagacagTGTTGGATCATATTCGTCCACAA

Table S5: Amplicon sequencing mapping statistics summary.

Sample	#Reads	#Aligned	%Aligned
NCI-H358_28D5 parental	84483	57443	67.994
clone #11	80484	60130	74.711
clone #12	86155	49963	57.992
clone #13	98367	47807	48.601
clone #14	61888	42654	68.921
clone #34	81374	62196	76.432
clone #36	75545	45556	60.303

References

1. Ryu SM, Hur JW, Kim K. Evolution of CRISPR towards accurate and efficient mammal genome engineering. BMB Rep. 2019;52(8):475-81.
